# *Molecular and Cellular Therapies*: New challenges and opportunities

**DOI:** 10.1186/2052-8426-1-1

**Published:** 2013-11-06

**Authors:** Xiangdong Wang, Dan Peer, Bryon Petersen

**Affiliations:** Shanghai Institute of Respiratory Diseases; Department of Respiratory Medicine, Fudan University Zhongshan Hospital, Shanghai, Sweden; Institute of Clinical Science, Lund University, Lund, Sweden; Department of Cell Research & Immunology, Laboratory of NanoMedicine, and the center for Nanoscience and Nanotechnology, Tel Aviv University, Tel Aviv, Israel; Department of Pediatrics, University of Florida, Children Health Research Institute, Gainesville, FL USA

**Keywords:** Molecular therapy, Cellular therapy, Disease, Journal

## Abstract

Gene therapy is suggested to be one of the most specific and efficient modulations for gene deficient diseases and extended to other diseases like cancer and inflammation, even though there are still challenges to be faced, such as specific and selective delivery, minimal to no toxicity, efficient metabolism, simplicity, and measurable efficiency. It is important to identify and validate drug-able disease-specific targets for molecular and cellular therapies, while it is equally important to have disease biomarkers to trace and define the biological effects of molecular and cellular therapies. The importance and significance of allostery in molecular and cellular therapies and “allosteric disease”, “allosteric effect”, and “allosteric drug” should be more carefully examined and validated. Cell therapy has been attracting an increasing amount of consideration in the development of new treatments for diseases. Molecular and Cellular Therapies (*MCT*) is a new, open-access journal, devoted to molecular mechanisms, preclinical and clinical research and development of gene-, peptide-, protein-, and cell-based therapies.

Welcome to the open-access journal titled Molecular and Cellular Therapies (*MCT*), a new international journal devoted to molecular mechanisms, preclinical and clinical research and development of gene-, peptide-, protein-, and cell-based therapies, including target identification and validation, safety, toxicity, efficacy, efficiency, pharmacokinetics, metabolism, formulation, delivery, pharmacovigilance, biomarkers, and new drug applications. *MCT* will integrate interdisciplinary fields such as genetics, cell biology, drug development, therapeutics, and new methods in biotechnology toward translational medicine. *MCT* aims to establish a scientific channel to translate the understanding of molecular and cellular therapies to clinical and medical applications, to improve the survival rate and quality of life for patients.

Alterations of gene frequencies were considered and proposed to be responsible for the development of diseases by Wright at the beginning of 2000s [1, 2]. Gene therapy is suggested to be one of the most specific and efficient modulations for gene deficient diseases and extended to other diseases like cancer and inflammation, although there are still challenges to be faced, e.g. delivery, toxicity, metabolism, simplicity, and measurable efficiency. The number of preclinical studies on molecular therapies recently increased and covered many aspects. For example, single-stranded antisense oligonucleotides (ss-siRNAs) had the therapeutic potency of about 100 fold more than unmodified RNA and about 30 fold- more than allele-selective inhibitors of mutant huntingtin expression in cells derived from patients [3, 4]. Such chemically modified ss-siRNAs could function under the need of Argonaute protein and through the RNAi pathway and provide allele-selective compounds for clinical development. This study showed that intraventricular infusion of ss-siRNA could selectively silence the mutant humtingtin allele throughout the brain in a mouse model. However, the metabolism, absorption, pharmacological dynamics and kinetics, protein binding, and elimination of ss-siRNA remain unclear. It is also not clear how ss-siRNA passes the brain, the tissue microenvironment, and the circulation and how it is penetrating into the cell. Furthermore, intracellular pharmacological profiles and associated metabolism of ss-siRNA may differ from other forms of RNA inhibitors. Another study demonstrated that ss-siRNA could target and inhibit mRNA both in vitro and in vivo by recruiting the RISC endoribonuclease Ago2 to cleave the mRNA [5]. About 50 phosphate analogs were suggested to be critical for the in vivo activity of ss-siRNA, rather than other forms like small interfering RNA due to the variation of human Ago2 activity, intrinsic potency, nuclease stability, and pharmacokinetics. An additional challenge of RNA inhibitors as one of the most attractive molecular therapies is how those inhibitors can identify and target the specific target cells within the tissue in order to maximize the therapeutic efficiency of such inhibitors. Nucleotide modifications have been considered to enhance metabolic stability and protein binding of the ss-siRNA to improve the rapid clearance of the oligonucleotides. On the other hand, the high protein binding of inhibitors will reduce the release and the biological efficacy of drugs within the tissue. An additional challenge is the delivery of oligonucleotides into the appropriate cells [6]. While even potent molecules need delivery strategies to overcome toxicity, specificity and metabolic degradation, there are numerous strategies currently being devised to address some of these challenges in cancer [7], inflammation [8], and viral infection [9].

It is also important to identify and validate drug-able disease-specific targets for molecular and cellular therapies, while it is equally important to have disease biomarkers to trace and define the biological effects of molecular and cellular therapies. Clinical bioinformatics was suggested to play an important and critical role in identification and validation of those disease-specific biomarkers, including omics technology, metabolic and signaling pathways, biomarker discovery and development, computational biology, genomics, proteomics, metaboliomics, pharmacomics, transcriptomics, high-throughput image analysis, human molecular genetics, human tissue bank, mathematical biology and medicine, protein expression and profiling and systems biology [5, 10]. We should focus more on the combination of clinical measurements and signs with human tissue-generated bioinformatics, understand clinical symptoms and signs of disease development and progress, and improvement of therapeutic strategies. In addition, attention should also be made to map relationships that integrate discrete elements that collectively direct global function within a particular -omic category, with clinical examinations, pathology, biochemical analysis, imaging and therapies. In order to develop better therapies, we should integrate new biotechnologies with the understanding of gene and protein functions, cell and organ dysfunction, and pathology, related to clinical signs, symptoms, findings, measures, prognosis and therapeutic effects.

The additional strategy of molecular and cellular therapies is suggested to balance the functioning regulation of genes, proteins, cells, and organs, rather than to simply “knock-in” or knock-out” a gene, develop an inhibitor for a protein. Nussinov and Tsai recently emphasized the importance and significance of allostery in molecular and cellular therapies and defined “allosteric disease”, “allosteric effect”, and “allosteric drug” [11]. Allostery is to modulate the single protein and the binding of an effector molecule at one site on the protein surface, which leads to changing the conformation of another site and then regulate protein activity associated with conformational and functional transitions in individual proteins. The first reaction is considered as allosteric site, while the secondary reaction can be the active or binding site. Allosteric mechanisms were proposed to be involved in the development of the disease and during the action of allosteric modulators (drugs). It was believed that new classes of drug therapies would come from allosteric drugs, rather than modifications of existing drug compounds, especially for many un-drug-able targets. The combinatorial allosteric drug regime should be the new strategy of molecular therapies for intractable, tenacious drug-resistant mutations to reach less toxicity but more efficacies. For example, a recent study investigated the effects of inhibitors targeting mutant genes as a differentiation therapy for cancer and found that the inhibitor could potently and selectively inhibit the tumor-associated mutant isocitrate dehydrogenases 2/R140Q in an allosteric manner [12]. Such inhibitor could induce differentiation of TF-1 erythroleukemia and primary human acute myelogenous leukemia cells, through the allostery and slow-tight binding of the inhibitor with mutant genes at the dimer interface. These mutations alter residues in the enzyme active sites and confer a gain-of-function in cancer cells, resulting in the accumulation and secretion of the oncometabolite (R)-2-hydroxyglutarate.

Cell therapy has attracted an increasing amount of attention in the development of new straties for the treatment for a variety of human diseases. There are a number of influencing factors that can decide the fate of stem cells. For example, the extracellular matrix (ECM) is a key component of the stem cell niche that can regulate stem cell behaviour, renewal or differentiation. Within the ECM there are different types of protein interactions that can trigger various cell responses via diverse sensing mechanisms and downstream signaling pathways [12]. The interaction between stem cells and ECM can be influenced by the intrinsic transcriptional or epigenetic state of a cell and the individual micro-environmental cues, including topography, ECM composition and stiffness, although the exact mechanism(s) by which ECM regulates the differentiation and renewal of stem cells remains unclear. As compared with stem cell transplantation, it is also possible to re-initiate the ‘reawakening’ pathways existed during embryogenesis for the repair of the injured tissue/organ. Non-myocytes were reprogrammed into cardiomyocytes with the expression of transcription factors (GATA4, HAND2, myocyte-specific enhancer factor 2C and T-box 5) and microRNAs (miR-1, miR-133, miR-208 and miR-499) that control cardiomyocyte identity, as a complementary approach for organ regeneration and repair [13]. Jiang et al. inserted a large, inducible single gene, XIST (the X-inactivation gene) into the DYRK1A locus on chromosome 21, in Down's syndrome pluripotent stem cells, to correct gene imbalance across an extra chromosome for Down's syndrome [14]. The XIST non-coding RNA was used to coat chromosome 21 and trigger stable heterochromatin modifications, chromosome-wide transcriptional silencing and DNA methylation to form a 'chromosome 21 Barr body', as a model to study human chromosome inactivation and creates a system to investigate genomic expression changes and cellular pathologies of trisomy 21 [15], free from genetic and epigenetic noise. It is one of the most successful achievements in the development of chromosome therapy. X inactivation is the transcriptional silencing of one X chromosome copy per female somatic cell, while exhibits considerable variation among species. It is evolutional to use X inactivation strategies in the development of stem cell therapies for inherited diseases. However, it is also important to understand the long-term arrangement from strict paternal stem cells inherited X inactivation, diversity of X inactivation strategies during cell differentiation, dominance, linkage, recombination, and sex-differential selection.

Protein-based drug development has been experiencing a vast array of opportunities as well as several challenges to overcome, even though it is well accepted that protein-based drugs have higher affinity and specificity from protein-protein interactions and are more effective to target and modulate the signaling pathways responsible for the occurrence of diseases. Brentuximab vedotin (SGN-35) is an anti-CD30 monoclonal antibody (cAC10) conjugated by a protease-cleavable linker to a microtuxbule-disrupting agent, monomethyl auristatin E. Brentuximab vedotin, has been approved as an effective treatment for relapsed CD30-expressing Classical Hodgkin and systemic anaplastic large cell lymphomas. Brentuximab vedotin was found to decrease cell proliferation, induce cell cycle arrest, trigger apoptosis of primary effusion lymphoma cells, inhibit tumor growth, and improve the survival of animals with primary effusion lymphoma [16]. Brentuximab vedotin and Trastuzumab emtansine have been recently approved for commercial distribution in the United States by the Food and Drug Administration, as next generations of clinically promising new antibody drug conjugate. However, the complexity of human disease highlights the need for alternatives that expand the therapeutic repertoire beyond this single class of proteins, indicating new opportunities and challenges for protein-based drugs. Next-generation therapeutics of protein-based drugs focuses more on the interaction between ligands and receptors, and should be combined in order to increase the efficacy and efficiency and reduce the toxicity and side-effect. These are only some examples for the vast data that is currently under pre-clinical and clinical testing.

There is an urgent and immediate need to create a forum to stimulate discussion and exchange of scientific findings and understandings of molecular and cellular therapies with a clear goal of treating diseases and improving the quality of patients. *MCT* is the journal focusing on the clinical application of gene-, peptide-, protein-, and cell-based drugs and keeping track of the wealth of new information related to the topic. We have the confidence to believe that the *MCT* will play an important, critical, and recognized role in understanding the molecular mechanisms of molecular and cellular therapies and developing the individual medicine and therapeutic strategy.

In conclusion, we as Editors of *MCT*, are delighted to welcome you to this new and exciting journal and thank the scientists who have agreed to publish in the journal. In setting up the journal, we owe an enormous debt of gratitude to all professors and scientists for their encouragement, support, comments, suggestions, and contributions. With great supports from our Section Editors, Associate Editors and Editorial Board Members, we deeply believe that *MCT* will be well-received both by preclinical, clinical and pharmaceutical scientists interested in molecular and cellular therapies and contribute to better outcome for understanding the diseases and developing new therapies. Involvement and contributions from a large group of scientists who work on molecular and cellular therapies are crucial to the success of the journal.
